# Emotional expression in human odour

**DOI:** 10.1017/ehs.2022.44

**Published:** 2022-10-10

**Authors:** S. Craig Roberts, Jitka Třebická Fialová, Agnieszka Sorokowska, Ben Langford, Piotr Sorokowski, Vít Třebický, Jan Havlíček

**Affiliations:** 1 Division of Psychology, University of Stirling, Stirling, UK; 2 Faculty of Science, Charles University, Prague, Czech Republic; 3 Institute of Psychology, University of Wrocław, Wrocław, Poland; 4 UK Centre for Ecology and Hydrology, Penicuik, UK; 5 Faculty of Physical Education and Sport, Charles University, Prague, Czech Republic

**Keywords:** olfaction, odor, fear, communication, smell

## Abstract

Recent work has demonstrated that human body odour alters with changing emotional state and that emotionally laden odours can affect the physiology and behaviour of people exposed to them. Here we review these discoveries, which we believe add to a growing recognition that the human sense of smell and its potential role in social interactions have been underappreciated. However, we also critically evaluate the current evidence, with a particular focus on methodology and the interpretation of emotional odour studies. We argue that while the evidence convincingly indicates that humans retain a capacity for olfactory communication of emotion, the extent to which this occurs in ordinary social interaction remains an open question. Future studies should place fewer restrictions on participant selection and lifestyle and adopt more realistic experimental designs. We also need to devote more consideration to underlying mechanisms and to recognise the constraints that these may place on effective communication. Finally, we outline some promising approaches to address these issues, and raise some broader theoretical questions that such approaches may help us to answer.

## Graphical abstract

**Figure:**
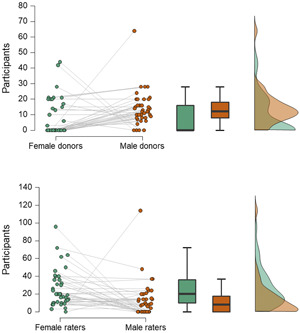

**Social media summary:** This review critically analyses the current evidence for communication of emotions through human body odour.

## Introduction

If we were to canvass the opinions of olfactory scientists on the question of whether there is an olfactory component in human emotional expression, even as little as 25 years ago, the answer would most likely have been a convincing ‘no’. This position has been dramatically transformed in the intervening years. Alongside a burgeoning interest in diverse aspects of olfactory functioning and the resulting recognition that the human sense of smell has been grossly under-estimated, there has been a surge of evidence to suggest that human emotions are expressed not just in the face and voice, but also through odour emitted from the human body. Indeed, studies indicate that emotion-associated changes in body odour can not only be detected by others, but can also induce physiological responses in these same others and affect their behaviour in contextually appropriate ways. In consequence, were we to pose the same question, today, we have no doubt that we would receive a very different answer.

In this paper, we will introduce the studies that have precipitated such a wholesale transformation in viewpoint. It is an exciting and persuasive body of evidence. However, it is also, as we have said, one that has developed recently and rapidly. It seems, therefore, also a timely opportunity to reflect on the implications, interpretation and limitations of the studies that have accumulated to date. We highlight some reasons for remaining cautious about the extent and nature of emotional communication in human odours and we outline some of the most pressing open research questions.

## Darwin's perception of odour and emotion

If scientists were unconvinced 25 years ago about the olfactory component in human emotional expression, the same can certainly be said of Darwin, 150 years ago, as he completed his book *The expression of the emotions in man and animals.* The possibility that emotional state might be expressed in odour, even in animals, does not appear to have occurred to him. Perhaps the closest he comes to this consideration might be his mention of nostril dilation in fearful horses, but he argues that this is the result of an association between fear and the exertions of rapid flight (Darwin, 1872: 130), rather than functioning to detect a source of danger. Notably, he does not consider the possibility of detecting a warning of danger from an odour produced by conspecifics – a phenomenon on which we focus in this paper.

He also comes close to the mark in his repeated discussions, at several points in the book, about the relationship between emotions and the erection of hair or other dermal appendages, such as the spines of the porcupine (p. 102). He views this as a manifestation of both fear and aggressive intent, whereby it serves to make an animal appear larger and thus a greater threat. For example, in the case of fear, he includes ‘the erection of the hair’ – what we now term piloerection – among a list of other defining features (e.g. trembling, pallor, cowering; p. 362). What he did not realise then, but we do now, is that piloerection is also involved in olfactory communication, at least in many species. In mammals, both sebaceous and apocrine glands open into the hair follicle, and it seems that piloerection increases the secretion of their odorous products, as well as enabling better air circulation on the skin surface (Flood, 1985). Piloerection appears to be integrally involved in emission of alarm odours in several species, such as the North American porcupine (Li et al., 2004), rat (Kiyokawa et al., 2004) and springbok antelope (Bigalke, 1972).

All this is not to say that Darwin was unaware of the importance of odours in communication – he clearly was. In *The descent of man* (Darwin, 1871), he includes odour-secreting skin glands as a product of sexual selection alongside weapons, ornaments and acoustic signals (e.g. p. 211). Indeed, he argues that odours are particularly involved in intersexual selection and he comments on numerous specific examples across various animal taxa. However, while he clearly saw continuity in characteristics between animals and humans, he was dismissive of the human sense of smell, arguing that we have inherited it ‘in an enfeebled and so far rudimentary condition, from some early progenitor, to whom it was highly serviceable, and by whom it was continually used’. It is, in short, ‘of extremely slight service’ (p. 18).

This view is certainly evident in *The expression of the emotions*. In total, he uses the word *odour* 13 times and *smell* 11 times. Perhaps unsurprisingly, the majority of these occur in Chapter 11, where he addresses contempt and disgust. There, he notes the way that the nose is centrally involved in the facial *disgust* expression, including wrinkling of the nose to contract the nostrils and often an accompanying sharp expiration of air in the form of a snort. However, he quickly moves on, dealing far less with how the sense of smell integrates with emotions and far more on how this facial expression has become stylised as a visual signal of disdain, to be triggered in social settings even in the absence of an unpleasant smell. In Darwin's view, then, the roots of the disgust expression certainly lie with odour and taste, but expression of the emotion itself is now predominantly visual.

## Changing views on emotional expression in odour

### Initial findings

Animals from ants (Wilson, 1975) to antelopes (Bigalke, 1972; Moy, 1970) have long been known to produce olfactory alarm signals that directly warn conspecifics of some form of current threat, such as an attack by a predator. Perhaps the story starts as early as the late 1930s, when von Frisch (1938) concluded that a substance released from the skin of injured minnows triggers fearful behaviour in others. We now know some of the compounds responsible for the behaviour, including hypoxanthine-3-*N*-oxide (Brown et al., 2000; Pfeiffer & Riegelbauer, 1978) and chondroitin fragments (Mathuru et al., 2012). In rodents, both short-term (Abel, 1991) and chronic (Valenta & Rigby, 1968; Zalaquett & Thiessen, 1991) stress can be discriminated in odour and can induce aversion responses towards previously preferred odours (Rottman & Snowdon, 1972; Zalaquett & Thiessen, 1991) or increases in other hiding and risk assessment behaviours (Kiyokawa et al., 2006). However, it was not until the beginning of this century that we began to document analogous effects in humans.

In the first such study, Chen & Haviland Jones (2000) collected odour on cotton pads worn under the armpit by odour *donors* (14 non-smoking women and 11 non-smoking men) on two consecutive days. On one day, a given donor viewed a 13 min excerpt from a funny movie; on the other, they viewed an excerpt showing people being menaced by snakes, insects and crocodiles (the order was counter-balanced across participants). The pads worn by women and men in each emotional condition (assumed to be ‘happy’ and ‘fearful’, respectively) were pooled as appropriate into one of four glass jars, which were stored in a freezer along with two control jars containing equivalent numbers of unworn control pads. One week afterwards, a double-blind testing procedure assessed whether people could identify happy or fearful odours. Each of 40 female and 37 male *receivers* (or ‘smellers’) did this in two ways. First, they completed four 3-choice tasks, where they were offered, in turn, the two female or two male donor jars plus the appropriate control. For example, in one of these four tasks, they were offered the happy, fearful and control jars from female donors, and asked to choose the jar that contained ‘odors of people when they are happy’. Then, they were given two 6-choice tasks, where they were presented with all six jars. In one, they were asked to select two happy odours from among the six jars; in the other, they had to select two fearful odours. When compared against chance, it was concluded that women could identify happy odours from both male and female donors, but men could do the same only for female odours. In contrast, both men and women could identify fearful odours from male donors, but not from female donors.

Continuing this line of research, Ackerl et al. (2002) asked 42 women to wear axillary pads and watch a frightening movie on one day and a non-frightening neutral one the next. Odour samples were then presented to female receivers in triangle discrimination tests; that is, smellers assess three samples and choose the one that is different, where two samples were worn during one movie and the third was worn during the other movie. Again, smellers chose correctly at rates better than chance, indicating a perceivable difference in odours collected under different emotional contexts. However, when asked to rate each odour on five characteristics (odour intensity, pleasantness, ‘smells like sex’, ‘smells like aggression’ and ‘smells like fear’), odours collected during the frightening movie were rated as more intense, less pleasant and more reminiscent of aggression, although, perhaps surprisingly, they did not differ from control odours in the extent to which they reminded smellers of fear (or of sex).

### Overview of recent evidence: scope, stimuli and response

Other studies followed closely behind, many using very similar experimental procedures. We do not attempt to review them all here, but instead direct the reader to previous reviews of emotion-related body odours (Calvi et al., 2020; Fialová & Havlíček, 2012) and a meta-analysis of studies that specifically examine the emotion of fear (de Groot & Smeets, 2017). For our purposes, it is sufficient to note that the most recent and comprehensive of these (Calvi et al., 2020) records 42 studies, including the two mentioned above, in which human body odours have been collected and presented to human receivers. The majority of these include anxious, fearful or stress odours. Fourteen induced fear in the same way as the initial studies, by exposing odour donors to horror movies. Seven other studies collected odours from donors while they participated for the first time in a tandem skydive, or who were traversing a rope course high above the ground. Another 10 collected odours from students about to undergo an examination, and three from participants undergoing the Trier Social Stress Test. In the latter four groups of studies (i.e. those not showing movies), the control condition overwhelmingly tends to be some form of physical exertion to induce sweating in the odour donors, usually on an ergometer such as a stationary bicycle (Haegler et al., 2010) or treadmill (Mujica-Parodi et al., 2009).

In contrast to this relatively standard set of core methodologies for emotional induction and odour collection, the outcome measures generated by these studies are more diverse (although several laboratories contributing multiple studies usually employ the same odour collection methods and outcome measures, creating families of similarly conducted studies). Thus, the fear literature often features outcomes using facial electromyography, EMG (de Groot et al., 2012, 2014, 2015a), or functional magnetic resonance imaging, fMRI (Mujica-Parodi et al., 2009; Radulescu & Mujica-Parodi, 2013), while a group of studies focusing on anxious odours present measures of the startle reflex (EMG of the eyeblink response, specifically) and/or electro-encephalography, EEG (Adolph et al., 2013; Lübke et al., 2017; Pause et al., 2009, 2010). Other studies use a range of measures, such as an emotional stroop task (in a study of aggression odour; Mutic et al., 2016), ratings of emotional expression in faces (Zernecke et al., 2011), a personality judgement task (of the competence and warmth of people seen in a video; Dalton et al., 2013), a risk game (Haegler et al., 2010) and even a measure of task performance in a simulation of dental procedures by trainee dentistry students (Singh et al., 2018).

Collectively, these studies contribute to an emerging picture of reliable and diverse responses to emotionally valenced odours in humans. In the following sections, we briefly outline the main findings of studies focussing on responses to odours collected from donors under various emotional states. Again, we do not attempt to review every emotion that has been addressed, but we first focus on fearful and anxious odours because these are by far the most studied, and then briefly address sad odours, because they have been studied in a rather different way.

### Responses to fearful odours

Compared with a control odour, fear odour elicits physiological changes including decreased cardiac parasympathetic activity, indicating increased stress (Ferreira et al., 2018), and electrodermal activity, indicating arousal (Endevelt-Shapira et al., 2018). In fMRI experiments, fearful odours trigger activation at the amygdala, responsible for emotional processing (Mujica-Parodi et al., 2009). When odours derive from female donors, this activation is more evident in female than male receivers, while male odours elicit similar activation in male and female receivers. These patterns perhaps reflect sensitivity to relative threat levels (Radulescu & Mujica-Parodi, 2013). Importantly, fear odour was not found to elicit any corresponding activation in the olfactory bulb, suggesting that these sex differences originate through emotional processing rather than through differences in perception (Radulescu & Mujica-Parodi, 2013).

Consistent with increased activation at the amygdala, fearful odours influence the processing of emotional facial expressions. Zhou and Chen (2009) showed that fearful (but not happy) odour made women more likely to judge an ambiguous facial expression as fearful, suggesting an altered threshold for facial threat detection. On the other hand, Mujica-Parodi et al. (2009) report that fearful odour facilitated greater accuracy of categorising expressions along a neutral–angry continuum, but in a way suggestive of sharpening judgements rather than reducing thresholds. Fearful odour increases the speed with which such facial judgements are made (de Groot et al., 2015a), and this appears to be emotion specific: fear odour increased the speed of recognition for fearful faces, but not for other negative facial expressions (anger, disgust; Kamiloglu et al., 2018).

In a series of studies, de Groot and colleagues (de Groot et al., 2012, 2014, 2018, 2020, 2021; Kamiloglu et al., 2018) also demonstrate increased medial frontalis muscle activity, characteristic of a fearful facial expression, when exposed to fearful odour. The response appears to be deeply rooted, because it was maintained while watching a video clip which presented a non-fearful situation (a woman chatting with a man; de Groot et al., 2014), indicating a degree of independence between visual and odour cues. However, it is worth noting that the effect was magnified when the audiovisual information was convergent with the olfactory cue (when the video clip depicted a man attacking a woman). Furthermore, the muscular response is also elicited when fearful odours are collected from individuals of a different ethnic and cultural group, suggesting a universal response (de Groot et al., 2018).

Another response to fear odour is increased effort towards sensory acquisition, which would be consistent with attempts to identify the source of potential danger. In a study comparing responses to fearful and disgust odours, de Groot et al. (2012) found that sensory acquisition responses depended on which odour participants were exposed to: sniff magnitude and eye scanning decreased after exposure to disgust odours, but increased following exposure to fearful odours (but see Endevelt-Shapira et al. (2018), where fearful odour led to reduced sniffing intensity). In another study using an interocular suppression task, which measures the extent to which the signal from one eye inhibits the other eye when presented with non-similar images, fearful odour shortened suppression times, indicating increased vigilance (de Groot et al., 2018). Fearful odour also increases attention to socially salient (angry) faces (Rubin et al., 2012) and enhances other facets of cognitive performance (e.g. in a word association task: Chen et al., 2006).

A further four studies have been conducted where the smellers belong to other species, including dogs (D'Aniello et al., 2018; Siniscalchi et al., 2016) and horses (Lanata et al., 2018; Sabiniewicz et al., 2020). These suggest that domestic animals can detect and respond in apparently appropriate ways to odour-mediated human emotional cues. For example, dogs exposed to human fearful odour have elevated heart rates and display more stressful behaviours compared with controls, and conversely are more willing to interact with strangers when exposed to ‘happy’ odour (D'Aniello et al., 2018). Similarly, Sabiniewicz et al. (2020) found that horses exposed to fearful or happy human odours showed differences in frequency of both head-lifting and touching a familiar person, in ways that suggest they were less relaxed in the presence of fearful human odour.

### Responses to anxious odours

As mentioned above, several studies explored the effect of anxious odours on the startle response, which occurs following some sudden stimulus and is part of the body's neural apparatus for defensive and escape behaviour (Lang et al., 1990). These studies universally find a larger startle response in the presence of anxious than control odours (Adolph et al., 2013; Lübke et al., 2017; Prehn et al., 2006), suggesting that chemosensory cues of anxiety prime and enhance defensive motor behaviour. This occurs despite there being no differences in explicit hedonic perception (i.e. odour pleasantness or intensity) between odour types. The effect is especially large in more socially anxious individuals (Pause et al., 2009), in whom there is also evidence for early processing bias towards anxious odour and increased allocation of neuronal resources (Pause et al., 2010).

Further studies indicate the variety of activational and behavioural effects induced by anxious odours. Anxious odours appear to moderate activation in brain areas associated with memory, social cognition and salience in the context of social ostracism (Wudarczyk et al., 2015), as well as areas monitoring anxiety in self (e.g. fusiform gyrus) and others (cingulate cortex), suggesting a mechanism for inducing empathy (Prehn-Kristensen et al., 2009). Anxious odours can also increase risk-taking behaviour (Haegler et al., 2010) and self-rated anxiety (Albrecht et al., 2011), and reduce vagal activity, consistent with a stress response (Rocha et al., 2018).

Several studies show that stress responses and emotional contagion mechanisms generated by anxious odours also influence facial processing, including enhanced allocation of attention towards faces (Adolph et al., 2010). Rocha et al. (2018) found that, although there was no effect on the speed with which participants were able to correctly categorise angry or happy faces, there was increased accuracy of facial expression recognition in the presence of anxious odour. Zernecke et al. (2011) had participants rate emotional faces along a neutral–happy morphing continuum; they rated ambiguously happy faces as less happy in the presence of anxious odour. In another facial expression recognition task, in an fMRI scanner, Wudarczyk et al. (2016) found that participants rated fearful faces as more fearful in the presence of anxious odour, and this corresponded to increased activation in cortical areas that facilitate the processing of socially relevant fearful stimuli. Finally, Pause et al. (2004) reported that while positive social evaluations were primed by happy faces in the presence of a control odour, this was reduced when exposed to anxious odour, possibly indicating a processing advantage for olfactory social cues over visual ones in ambiguous situations.

### Responses to sad odours

In contrast to the literature on fearful or anxious odours, described above, in which studies universally focus on axillary odour, there is a small but interesting group of papers which addresses the possibility that chemical communication can also occur through tears. Although it has been argued that emotional crying is a uniquely human phenomenon (e.g. Gračanin et al., 2018), and certainly it is by no means widely observed in other species, there is some evidence for emotional communicative effects in the lachrymal gland secretions of some rodents. Secretions from the harderian gland, found in the orbit of some rodents and thought to be an accessory to the lachrymal gland in some species, inhibit aggression and appear to induce appeasement in blind mole-rats (Shanas & Terkel, 1997). A variety of effects of lachrymal gland secretions have been reported in mice, including stimulating aggressive behaviour by other males (Thompson et al., 2007), co-ordination of courtship behaviour between adults (Kimoto et al., 2005), and when produced by juveniles, inhibiting sexual behaviour by adult males (Ferrero et al., 2013).

In humans, however, it seems likely that any social communicative function of tears would be associated with sadness. This was the starting point of the study by Gelstein et al. (2011), who examined responses to tears elicited by people watching sad movies. Surprisingly, exposure to sad odour in tears (as compared with a control saline solution) did not alter attributions of sadness in emotionally ambiguous faces, nor did it alter self-ratings of positive or negative mood. In contrast, it did have significant effects on sexual interest: faces appeared less sexually attractive and levels of both sexual arousal and salivary testosterone decreased in men smelling female emotional tears. Although an attempt to replicate this study failed (Gračanin et al., 2017), the original finding was subsequently defended robustly and, indeed, it was claimed in this defence that some of the replicated data supported the initial finding (Sobel, 2017). A further study by an independent group replicated the finding on reduced testosterone levels in men following exposure to sad tears produced by women (Oh et al., 2012).

## Reasons for caution

The evidence that we have reviewed above indicates that human body odour is altered when experiencing different emotions, and that exposure to these emotional odours can elicit a range of psychological and physiological changes in other individuals. In combination, these two elements constitute the key requirements for olfactory communication of emotional state, whether actively or passively, and perhaps also thereby the transmission of emotions among individuals in a social group or network.

Since it is now based on nearly 50 studies, this recent literature would seem to paint a very clear picture of a species which is much more sensitive to olfactory cues of emotion than previously believed. As researchers in human social olfaction, we are enthusiastic and excited by this shift in view. Despite this, however, we believe that there remains a need for considerable caution when we take these laboratory findings and speculate on the importance of emotional odours in everyday life.

The findings reviewed above strongly suggest that body odour emissions alter with emotional state. They further suggest that it is *possible* for humans to detect and respond to emotional odours, at least under laboratory conditions, but whether we *actually* do so in real life remains to be robustly tested and demonstrated. Such concern over correspondence between laboratory findings and the real world is, of course, not unique to emotional odours; it is a common concern across many fields of psychological science. Nonetheless, in the following sections, we highlight the particular issues facing the study of emotional odours (and, to some extent, studies of human body odour in general) which place limits on our ability to extrapolate to the real world. These include methodological choices regarding both odour stimuli and smellers in emotional odour studies, the question of effect size and real-world relevance, and the need for progress on our understanding of underlying mechanisms.

### Issues concerning stimuli

Experimenters typically apply a series of design decisions about the stimuli to increase the chance of detecting an existing effect. While controlling for sources of additional variability in the data is to be expected in scientific studies, these decisions can be extensive in scope and each reduces the extent to which the presented stimuli are representative of everyday experience.

First, experimenters standardise the odour stimuli as far as possible. Donors must belong to a specified age range and sometimes sexual orientation, since both factors may influence their perceived odour (Martins et al., 2005; Mitro et al., 2012). Furthermore, donors must abide by specified lifestyle restrictions to reduce the influence of background odour. Usually this involves two or more days of refraining from eating foods that influence odour (e.g. garlic, Fialová et al., 2016), from drinking alcohol or even coffee (Mutic et al., 2016), and from smoking (Fialová et al., 2018) or being around smokers. Donors may even be asked to avoid excessive exercise for several days (e.g. de Groot et al., 2012) or going to the swimming pool or sauna (Mutic et al., 2016; Wudarczyk et al., 2015). In addition, many studies ask participants to wear clothing which is pre-washed using non-scented products, and almost all prescribe that they refrain from using any personal fragrances, deodorants or scented shower products. This is not only because such products reduce the salience of the underlying body odour itself; they also interact with the odour in a way that influences perception of the donor's personal characteristics, including personality, attractiveness and distinctiveness (Allen et al., 2015, 2016; Sorokowska et al., 2016).

**Figure 1.**
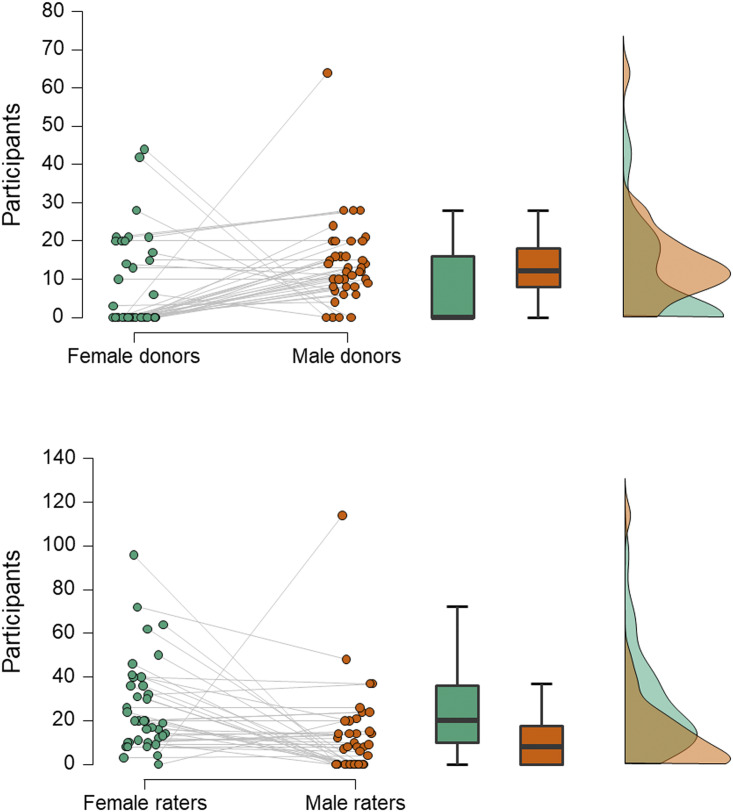
Numbers of male and female participants acting as odour donors or receivers. Data are from 40 studies of axillary odours conducted between 2000 and 2018. In the raincloud plots (left), jittered dots represent females (green) and males (orange) in individual studies (which are connected by grey lines). Boxplots show median (thick line), interquartile range (box) and minima and maxima (with error bars); the density plot shows data distribution. Compared with men, women less often act as donors (upper panel; Wilcoxon test, *W* = 447.5, *z* = 2.98, *p* = 0.003) and more often as receivers (lower panel; *W* = 108, *z* = 2.92, *p* = 0.004).

Second, experimenters more often choose to use men as odour donors and women as smellers (Figure 1). While such a design is sometimes used in studies of mate selection (Ferdenzi et al., 2020), where it is thought that heterosexual females assess potential partners more carefully than heterosexual males, and because smell is considered especially important to women in this regard (Havlicek et al., 2008), this directional sequence from male donor to female smeller would seem less applicable to communication of emotion: both the sender and receiver of any emotional signal could be of either sex. As the reason for selecting men as donors is often unstated, we can only infer that it may be because men have larger apocrine glands and produce more intense axillary odour than women (Doty et al., 1978), thus making it more likely to find hypothesised effects (although this would not be an explanation for those studies that ensure odours are presented at, or just below, threshold concentration). Alternatively, it could be to avoid menstrual cycle related changes in women's odour (Havlíček et al., 2006; Singh & Bronstad, 2001), which could potentially interfere with the hypothesised effect.

Third, experimenters sometimes deliberately seek extreme circumstances to collect stimuli. Recruiting as donors people about to jump out of an aeroplane for the first time is certainly a novel methodology (Mujica-Parodi et al., 2009), and the intensity of the experience probably enhances the chance of capturing a fearful odour, but it is unquestionably extreme and not easily comparable with most other context manipulations (a separate issue is that it may also be tinged with other emotions, such as excitement and relief). In a different way, Zhou and Chen (2011) also took steps to ensure that they obtained extremes of emotion. Participants watched fearful, funny or sexually arousing videos, each day, for three consecutive days; immediately after each, they assessed how much that video evoked each emotion. The experimenters then chose to use only the odour sample collected during the most appropriately evocative video. For example, they chose to use as a fearful odour only the sample that was collected during the scariest of the three movies (as rated by a given donor), and they discarded the other two samples. Again, such decisions are reasonable if the aim is to demonstrate whether it is *possible* for odour to communicate emotions, but they also place limits on the generalisability of the study's conclusions (unless, perhaps, if all responses to emotional signals are always dose-invariant, as one recent study suggests could be the case: de Groot et al., 2021).

Unlike the points above, which each relate to ways of increasing the chance of finding effects, a final issue is about whether stimuli adequately or accurately capture the putative emotional odour. As we have seen, stimuli are collected from odour donors in advance of presentation to smellers – for example, on an axillary pad while watching a frightening movie for a certain amount of time. This time is often 20 min (e.g. Zhou & Chen, 2011) while Haviland-Jones et al. (2016) compared a 24 min period with a ‘short’ collection time of 12 min. One problem with this method in general is that the sample, when presented, is a poor reflection of the real changes in odour that have taken place during the time in which it was collected (a similar point has been made to use of static facial images in visual communication: Jack & Schyns, 2015). These changes are almost certainly dynamic, increasing and then decreasing in intensity; they almost certainly also change in quality (i.e. the relative proportions of constituent volatile compounds), since we would expect a true alarm signal to occur within the first seconds or few minutes of a typical collection period. The collected odour sample, then, is a static amalgam of this dynamic whole, presented all at once to the smeller. We simply do not yet know whether the second is an effective substitute for the first. Nor do we know how rapidly the effective components of the emotional odour are generated.

### Issues concerning smellers

**Figure 2.**
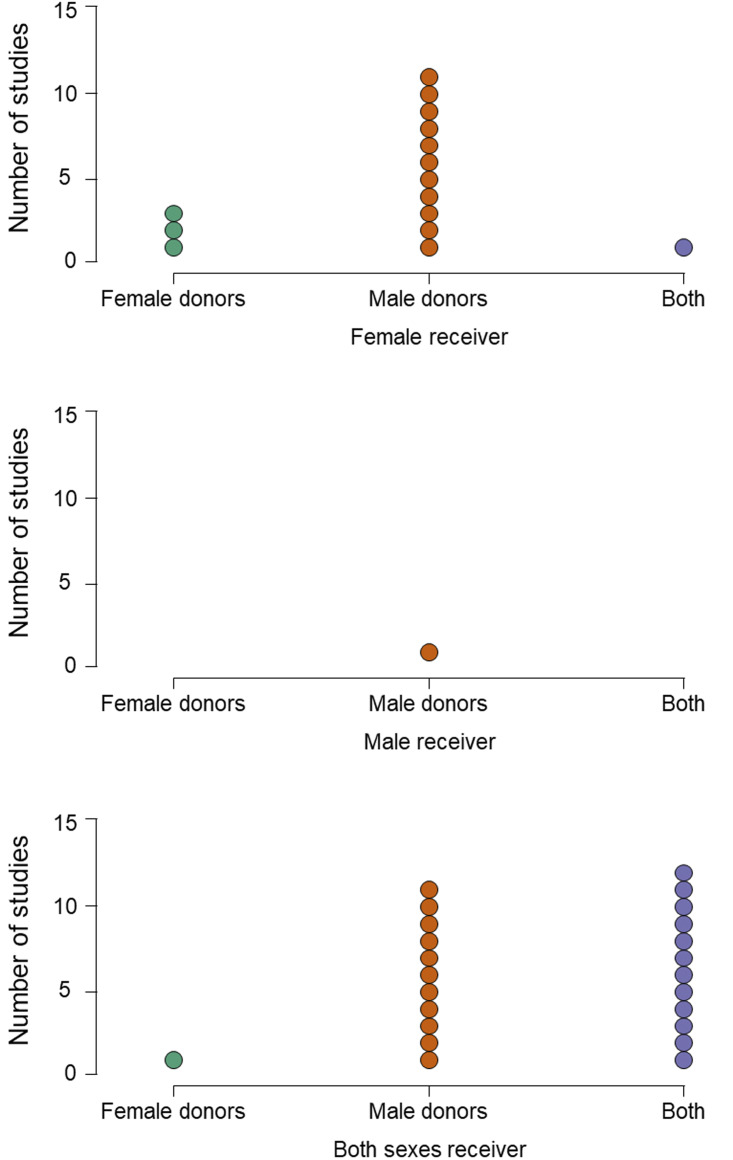
Imbalance in study design. Panels show the number of studies in which receivers are only female (upper), only male (centre) or both males and females (lower). Within each receiver type, studies present odours from only female donors (green dots), only male donors (orange dots) or from both (purple dots).

In similar ways to stimulus collection, most studies also maximise the chance of detecting their hypothesised effect by applying several inclusion or exclusion criteria to potential smellers. For example, in all of the reviewed studies, smellers were healthy, with no respiratory disease and with normal olfactory function; often those with any allergies are also excluded (e.g. Kamiloglu et al., 2018). In most, the participants’ olfactory function was pre-assessed using one or other form of screening test (e.g. Sniffin’ Sticks threshold test) to ensure that they had a functional sense of smell. Smellers also tend to be young, avoiding older participants whose smell function may be declining (Sorokowska et al., 2015). In almost half of the studies, smokers were excluded, presumably on the basis they may not perform as well. In a number of studies, only women not using hormonal contraception were included (Mutic et al., 2016; Pause et al., 2010), either for the same reason or to standardise variability in olfactory functioning related to menstrual cycle stage (Martinec Nováková et al., 2014) – for example, in one study, female smellers were tested only in the luteal phase of their cycle (Mutic et al., 2016). Finally, as we have seen, experimenters more often recruit women as smellers (Figure 1), and only one of 40 studies used a design in which only men were receivers, compared with 15 where only women were (Figure 2). Calvi et al. (2020) suggest that this pattern is typical because women tend to out-perform men in olfactory tasks (Sorokowski et al., 2019) or are more involved with emotional interactions in social settings (see also de Groot et al., 2012).

Furthermore, presentation of odours to smellers is conducted in a way that ensures odours are optimally detectable. Thus, odours are delivered via tubes to the smeller's nose (e.g. from an olfactometer), in devices held directly under the nostrils or in other containers lined up on the lab bench from which smellers are asked to inhale deeply. While these approaches ensure detection, the real-life distance between an individual who emits a given emotionally laden odour and the receiver who smells it will usually be more than 2 cm (cf. de Groot et al., 2015b; Zernecke et al., 2011; Zhou & Chen, 2011). What is a more realistic distance? Clearly this will vary considerably, but cross-cultural data suggests that preferred inter-personal distances between strangers are 135 cm, compared with 92 cm between acquaintances and 32 cm between close people, with women usually preferring larger distances than men (Sorokowska et al., 2017). To our knowledge, only one study has tested emotional effects over even the smallest of these mean distances. Singh et al. (2018) exposed dental students to anxious odours when doing a performance test, and the t-shirt containing the anxious odour was ‘worn’ by a dummy patient, thus simulating a semi-natural presentation. We applaud this study, although even here, the conditions were favourable compared with many real-life settings where temperatures may be lower, affecting compound volatility, or which may have turbulent air currents that interfere with odour detection.

### Effects sizes are small to moderate

In their meta-analysis of responses to fearful odours based on 19 available studies, de Groot and Smeets (2017) estimate the overall (Hedge's *g*) effect size to be 0.36 [0.31, 0.41], indicating a small to moderate effect according to conventional rules of thumb (Cohen, 1992). We agree with them that this effect size seems at odds with a general perception of poor olfactory ability in humans compared with other species. Indeed, some researchers would suggest that, regardless of conventional standards, an effect size of this magnitude should be considered of practical significance and likely to have powerful influence (Funder & Ozer, 2019).

On the other hand, it is worth remembering that this mean effect size was achieved following the implementation (in the original studies) of all the methodological procedures and restrictions described in the previous two sections, each of which are designed to facilitate the detection of effects. The interesting question is how accurately this average laboratory effect size reflects what occurs in the smelly milieu of the real world. While psychological effects do tend to generalise from laboratory to the field, this varies across subfields and social psychology is one of the least generalisable (Mitchell, 2012). Thus, we must remain cautious in our interpretation of the effects of emotional odour on behaviour while we await studies in less carefully controlled settings or with fewer restrictions on odour donors and smellers.

### We know little about mechanism and that's a problem

The production of specific emotional cues probably involves a complex chain of physiological processes occurring both beneath and at the skin surface. We do not yet know the identity of the key volatile molecules responsible for emotional cues, nor do we yet fully understand the pathways involved in production of even relatively stable axillary compounds that we do know are responsible for characteristic axillary odour, such as the abundant sulfur compound 3-sulfanyl-3-methyl-hexan-1-ol (also known as thiol). However, we can illustrate the likely chain of necessary steps in the production of an emotional axillary cue by summarising the comprehensive pathway that was recently proposed by Natsch and Emter (2020) for thiol.

First, a precursor (3-methyl-2-hexenoyl-CoA) is hydrolysed to form 3-methyl-2-hexenol, which is then converted to a glutathione conjugate by the enzyme glutathione-*S*-transferase. The conjugate is transported across a plasma membrane from the cytoplasm of a secretory cell into a secretory vesicle by the transport protein ABC11. Here, the terminal glutamine residue is removed by the enzyme γ*-*glutamyl transferase 1 to form a Cys-Gly conjugate. The secretory vesicles are then released into the secretory ducts of the apocrine glands, and subsequent vesicle rupture finally brings the conjugate onto the skin surface. However, the process is not yet complete, because the conjugate is odourless; release of the odorous volatile compound requires further metabolic steps by certain subsections of the dermal microflora. Thus, the conjugate is actively carried by a transporter protein across the bacterial membrane, where it is cleaved by a dipeptidase enzyme, and then a *β*-lyase releases the sulfur compound. Owing to its low molecular weight, the thiol compound can then passively diffuse across the bacterial cell membrane onto the skin surface. Finally, evaporation from the skin surface enables detection by the nose as an axillary odour.

We describe the pathway in some detail to make three points. First, its elucidation is the work of several laboratories over many years, meaning that we will probably have to wait some considerable time before we can similarly describe the production of any emotional odour. Second, emotional pathways may be even longer and more complex than described above, because we may assume that there is an additional chain of physiological (i.e. neural, hormonal or metabolic) responses that precede the activation of an analogous pathway for an emotional odour; for a response to a fearful odour, for example, the pathway would probably involve the sympathetic nervous system. Third, it remains unclear how long this process takes from beginning to end, but it is certainly not an instantaneous reaction.

There is a point of view that understanding mechanisms is not as important as exploring the functional aspects of a phenomenon: we can remain agnostic about mechanism while we investigate and describe function, on the understanding that we will fill in the gaps in our understanding at some later point. For example, behavioural ecologists have been doing this for many years in an attempt to model natural selection in the absence of detailed knowledge of the relevant genetic processes (Grafen, 1984). Yet as far as understanding emotional odours is concerned, we are now reaching a point where uncertainty over mechanism may constrain further progress. At the very least, more understanding of mechanism might prevent us from going down blind alleys based on unrealistic assumptions about how such odours regulate behaviour.

Perhaps this applies especially to the third point. Clearly, the delay between the onset of a specific emotion, the production of a related odour at the skin surface and subsequent detection of the odour by another individual will define the limits of its functionality as a means of communication. Notwithstanding the evidence for apparently appropriate behavioural and physiological responses to fearful odours that we reviewed above, and the potential survival benefits of such responses, one must question the utility of a fearful odour if it arrives too late to save oneself from harm. Its utility is undermined anyway if alternative ways to detect the threat, for example via visual or auditory cues, are more rapid and reliable. In short, if we neither have a clear understanding of the chemical structure of a fearful odour, nor of its synthesis and hence schedule of emission in relevant contexts, then we risk making unfounded assumptions regarding its adaptive value.

## The way ahead

As we have outlined so far, the past two decades have seen a rapid burst of studies that collectively provide convincing evidence that humans have the capacity to both emit odours that contain information about emotional state and to detect and respond to these odours in appropriate ways. However, we have also pointed out some shortfalls in this evidence. In this section, we therefore describe some of the key outstanding lines of enquiry which we believe warrant particular attention in the near future.

### Improving ecological validity

Perhaps the most pressing need in future studies is to increase the degree of ecological validity in emotional odour research. We do not mean that every study should do this, but at least some studies should test effects in more realistic ways. In particular, studies should (a) place fewer restrictions on donor lifestyles, (b) have the same number of men and women as both donors and smellers, (c) place fewer restrictions on the selection of smellers and (d) present odours at distances and in environmental contexts that are more representative of social interactions.

We are aware that designing studies like this, and doing it well, is not a trivial task. We have already highlighted the study by Singh et al. (2018) who, by having dummies (dental ‘patients’) wearing t-shirts impregnated with specified odours, were able to deliver the odour to the smellers (dental students) in a realistic manner for the given context. In the case of less context-specific scenarios, studies could alternatively adapt the kind of approach used by Gaby and Zayas (2017), who tested responses to people's odour in a procedure that mimics a typical interaction between strangers.

Studies with more realistic designs should enable us to obtain an accurate estimate of the effect of emotional odours on human behaviour. It will be fascinating to see how this might compare with, for example, the effect size calculated for responses to fearful odours in de Groot and Smeets’ (2017) meta-analysis of laboratory studies. While it might be expected that effect sizes in more realistic conditions will be smaller, this is by no means always the case (Mitchell, 2012) and it may even be that enhanced realism of presented scenarios facilitates the release of more pronounced effects.

Finally, whatever the outcome, we should bear in mind that even the most realistic of scenarios can only inform us about the investigated effect in the tested population. It is possible, perhaps likely, that studies return lower effect sizes if they are conducted among people from Western, Educated, Industrialised, Rich and Democratic countries (WEIRD, Henrich et al., 2010), not least because WEIRD people are also ODD – that is, they are relatively old, and live in deodorised and desensitised conditions (Roberts et al., 2020a).

### Understanding poor performance in emotional labelling

Conscious perception is not necessary for appropriate responses and, indeed, it may interfere with spontaneous processing (Li et al., 2007); for this reason, many researchers present odours at subthreshold levels. Nonetheless, it is interesting that people appear to be rather poor at tasks such as labelling emotional odours. For example, Chen and Haviland-Jones (2000) found that 54% of men and 53% of women could identify the fearful odour in a three-way choice between a male fearful odour, a male happy odour and a no-odour control. Although these were significantly better than chance (33%), it still means that almost half of smellers could not correctly identify the odour, even under lab conditions and with the same kinds of restrictions on donors and smellers described earlier. Success rates were even worse for fearful odour from female donors, at chance levels (35 and 30% for male and female smellers, respectively).

Such performance scores when labelling emotions in odour stand in contrast with comparable performance levels for facial expressions: often over 80% in tests where judges see faces from their own population, despite there usually being twice as many incorrect alternatives than in odour studies (Ekman et al., 1987; Elfenbein & Ambady, 2002). We think there are two likely explanations for why humans appear to lack comparable skills in labelling emotional odours to those for faces. The first is that emotional odour expression is simply less important than emotional facial expression (even if it was important in our evolutionary past). For example, it might be important only in limited and specific contexts, such as in the dark when visual expressions are inaccessible, or only among very intimately bonded pairs (cf. Zhou & Chen, 2011). An alternative possibility is that humans have capacity for the same level of skill as in faces, but we see low odour identification rates because, again, we tend to test in ODD populations where people do not have sufficient opportunity to learn or maintain skills in olfactory perception (Roberts et al., 2020a).

Although it does not distinguish between these ideas, evidence that people improve with practice is especially consistent with the latter suggestion. Such evidence comes from the study by Haviland-Jones et al. (2016), who concluded that about 50% of people are consistently accurate ‘super detectors’, about 33% are above-chance level detectors and only 18% are non-detectors who are effectively anosmic for emotional axillary odours. The basis for these classifications was performance in an emotional labelling task where ‘super detectors’ approached 80% accuracy, on average, and those in the detector group approached 40% accuracy. The former rate may seem especially impressive, but it is worth noting that this was reached only after 15 trials with some implicit training between each trial; success rates in the first trial were at chance levels, even in so-called ‘super detectors’.

### Elucidating the underlying mechanisms

Smeets et al. (2020) describe social communication via human axillary odour as a ‘black box’, a system that we can describe in terms of its inputs and outputs without knowledge of its internal machinery. In their study, they examined more than 1600 individual volatile compounds recorded from axillary sweat of a sample of young men who watched emotional movie clips for 30 min over three sessions, yielding samples for each participant of fearful, happy or neutral odour. They reported differences in patterns of volatile compounds between the three odour types, such that fearful odour contained fewer esters and cyclic molecules, and more linear aldehydes and ketones, compared with neutral odour. Happy odour contained two subclusters of participants, one where the chemical profile of their happy odour resembled that of their fearful odour, the other where their happy odour more resembled their neutral odour.

Two other studies (Preti et al., 2019; Williams et al., 2016) have examined chemical composition of breath odour in relation to emotional content, reporting the potential importance of the compounds isoprene, acetone and dimethyl sulfide. In Preti et al.'s study, these three compounds were elevated during Trier Social Stress Tests. Interestingly, levels were not elevated in saliva, suggesting that they originate from metabolic processes elsewhere. The evidence suggests that acetone is associated with the release of adrenaline and its effect on glucose production by the liver, while the other two compounds are associated with cortisol release. In their study of cinema audiences, Williams et al. suggest that isoprene levels are mainly indicative of muscle activation during movement, but also found increased production during certain emotionally arousing scenes in which viewers remained seated, and like Preti et al., they suggest that this could be related to emotion-moderated cortisol increase. Williams et al. further found associations between scenes with specific emotional content (including injury and comedy) and some other volatile compounds. Although these were interpreted as being compounds predominantly from breath, Williams et al. measured ambient air and thus they probably also included axillary odour changes.

As this is the sum of what we currently know about the chemistry of emotional odours, it is clear that much work remains to be done. However, we are optimistic about the potential for progress based on the technique that Williams et al. (2016) introduced in their cinema study. They used proton-transfer-reaction time-of-flight mass spectrometry (PTR-TOF MS), which has capacity to measure change in airborne volatiles on a continuous basis.

This feature radically alters the ability of researchers to investigate emotional odours, introducing a novel set of experimental approaches to study olfactory communication at the individual level (Roberts et al., 2020b). This is because researchers can now measure real-time change in odour emitted from undisturbed individuals or groups as they experience changing emotions. As we have described, previous studies might show a 20 min movie clip to capture a single odour sample at its conclusion, an amalgam of the changing emotions over that period. Now, we can measure and chemically analyse how the odour changes from scene to scene. For example, preliminary evidence shows that changes in axillary odour can occur relatively rapidly: emission rates of acetone and butanone sampled directly from the axillary skin surface altered within 2–3 minutes after the initiation of frightening scenes (Roberts et al., 2020b). Not only this: we can also direct the same odour stream to the nose of other individuals, enabling us to investigate how changing odour chemistry of the donor affects co-occurring real-time physiological or behavioural responses in receivers.

The technique has a further advantage over traditional analytical procedures. In contrast to a pad worn under the axilla during an emotional event, or a swab taken at the end of it, both of which capture non-volatile compounds from the skin surface, PTR-TOF-MS measures only airborne volatiles. This means that researchers record only compounds that might be accessible to the nose, narrowing the search for the active compounds within the odour.

Limiting the search to volatiles is especially useful because PTR-TOF-MS records the changing concentrations of hundreds of compounds, generating large and complex datasets. Optimism about future progress is enhanced by new statistical approaches that can aid analysis of these datasets. One such approach is not to analyse the changing multi-component odour trace compound by compound, but rather holistically, to identify groups of compounds that co-vary in their temporal expression and might be related to a given event or state. An example of such a technique is positive matrix factorisation, which is routinely used in fields such as atmospheric chemistry (Langford et al., 2010). Applying this to human odour, the technique could be used to identify the suite of odours that alter at the onset of a given emotional event (Roberts et al., 2020b).

### Are there specific chemical signatures for basic emotions?

The adoption of techniques like PTR-TOF-MS and positive matrix factorisation should enable us to answer a range of critical questions, the most important of which may be the same kinds of questions that remain disputed and controversial in the wider field of emotion research and the study of emotional facial expressions (Barrett et al., 2011; Nelson & Russell, 2013). One such example relates to the concept of basic emotions, which are thought to have direct adaptive value in specific recurring contexts (Ekman, 1992). A key component of Ekman's proposal that anger, fear, enjoyment, sadness and disgust are basic emotions is that they have distinctive universal signals; that is, they each have a characteristic facial expression that can be described in terms of activation patterns across the facial musculature and that are reliably recognised by people from the same and different human cultures (Ekman & Friesen, 1971).

The literature we reviewed above suggests the possibility that there are also changes in human odour during emotional experience, but we cannot be sure of the extent to which these changes are discrete and characteristic of a given emotion (recent research suggests that even facial expressions may not be as discrete as previously thought: Barrett, 2017). We can only currently draw inferences from studies that compare behavioural outcomes of exposure with specific emotional odour stimuli. Here, the evidence is mixed. Some studies have found that fearful odour elicits responses that are directly appropriate only for a frightening situation. For example, de Groot et al. (2012) reported distinct behavioural responses to fearful and disgusting odours from the same odour donors. Furthermore, relative to neutral odour, exposure to fearful odour induced the activation of muscles responsible for producing a fearful facial expression and faster responsiveness to other faces displaying a fearful expression, but not to angry or disgusted facial expressions (Kamiloglu et al., 2018). Similarly, Silva et al. (2020) found faster responses to fearful facial expressions after exposure to fearful odour, but they did not see similar effects after exposure to disgust odour, suggesting that such effects are not simply driven by negatively valenced odours. On the other hand, other studies suggest responses that are not specific to the single target emotion. For example, fearful odour elicited faster responses to angry faces (Rubin et al., 2012) or to both happy and neutral faces as well as fearful ones (de Groot et al., 2018), while anxious odour also influences the perception of happy faces (Zernecke et al., 2011).

This mixed evidence leaves open the critical question about whether there are chemically distinct emotional odours that are reliably produced in specific situations and only in these situations. Distinct responses to specific contexts, such as that observed by Kamiloglu et al., would appear to be convincing evidence for discrete chemical signatures. In contrast, non-specific responses, such as those observed by Zernecke et al., do not settle the argument either way: they might be evidence against discrete signatures, but could also arise simply because of interdependence between different emotional states. If distinct signatures do exist, and have adaptive function, then they should likely be produced in response to the same environmental stimuli as those that produce analogous facial expressions, and it should in time be possible to describe them in terms of their distinct signatures of volatile organic compounds.

### How universal is their effect?

Another question for the future is whether odours associated with different emotions are reliably recognised as such by individuals in other social groups and cultures. The answer will be insightful not only with reference to the extent that odours are involved in emotional communication between individuals, but also regarding the odour's evolutionary origins.

As a first step, simply determining whether given odours are reliably recognised by other individuals is not as straightforward a task as might first appear. As reviewed above, many (or most) people have difficulty in accurately labelling or identifying a specified emotional odour, and because appropriate responses are not contingent on this, researchers often present odours just below threshold concentration. A problem with this is that there may be significant variation in response between individuals, not least owing to differences in olfactory sensitivity but also dependent on aspects of emotional state, such as individuals with social anxiety responding more strongly to anxious odours (Pause et al., 2009). Another issue is that observed responses may be very different, even apparently opposite, depending on the task or context presented to participants by experimenters. This point is illustrated by de Groot et al. (2017) with the example of cautious vs. risky behaviour, where participants can appear to both expend more effort towards accuracy in distinguishing indicators of threat (Chen et al., 2006) and yet make more risky decisions (Haegler et al., 2010) following exposure to fearful odour.

If within-population responses are dependent on individual and contextual factors, it is likely that they will be still more variable when compared across different human populations. Satisfactory design of robust tests is therefore critical if we are to tackle the important question of inter-cultural responsiveness to emotional information in body odour. To date, this has been addressed by only one study, whereby de Groot et al. (2018) used tried-and-tested non-verbal methods, notably facial EMG, to measure responses of Chinese and Dutch women to fearful, happy and neutral odours from a sample of Dutch men. They found broadly equivalent and emotion-relevant responses in both samples of women, suggesting that perception of these emotional odours is population independent. Although further studies are needed, this study indicates that both the chemical nature of emotional odours and the induction of appropriate responses to them may be species-wide traits. Employing emotional odours from individuals of East-Asian origin would be particularly informative. These populations are characterised by widespread ABCC11 mutation (over 95%), which makes their axillary odour of considerably lower intensity (Martin et al., 2010). Whether this mutation also affects emotional odours remains to be determined.

### Are there prototypic odours?

A universally recognised emotional odour would suggest a conserved chemical signature that is shared across populations, and the presence of universal and apparently adaptive responses would strongly indicate the potential for emotions to be communicated through odour. A further unanswered question then is whether these odours might have undergone a process of ritualisation over evolutionary time, in a process parallel to that proposed in the generation of facial expressions of emotion (Shariff & Tracy, 2011). According to this view, facial expressions of emotion have their roots in functional responses to the situations that evoke specific emotions, such as the wrinkled nose and nasal constriction of the disgust expression which limits intake of the offensive odorants towards the olfactory epithelium. However, over evolutionary time, these expressions can become more visible, exaggerated and distinctive in order to make social communication of the emotion more effective, and they may become ritualised even to the point where they are displayed in contexts where the original stimulus is absent or abstract (e.g. a disgust expression to a non-odorous stimulus; see Lorenz, 1966).

To what extent, then, might odours have followed a similar process? If there are direct fitness benefits to both sender and receiver in emotional odour communication, then selection could potentially have acted to make this information flow more efficient and reliable. It is theoretically conceivable that we could develop a ritualised representation of emotional state in odour, as in facial expressions. In other words, we could produce an exaggerated, prototypic version when we communicate with young children, or in social vs. solitary situations, or when forms of communication using other sensory modalities are compromised. It will be interesting to test these ideas, but again, this awaits much better knowledge of the underlying chemical mechanisms.

### Are they signals or cues?

In the previous section, we hypothesised about the existence of prototypic emotional odours, but it seems more likely to us that such ritualisation may be limited, or altogether prevented, by constraints that are unique to olfactory communication. We suggest that such constraints derive from the metabolic processes involved in production of characteristic odours.

For example, if the characteristic chemical structure of a fearful odour originates in microbial action on breakdown products of hormones produced in the acute stress response, it would seem unlikely that hallmarks of a putative prototypic odour – manufacture of these breakdown products in the absence of the stress response itself, or their amplification in the presence of a low-level response – could exist. Instead, the fearful odour would be produced only in fearful conditions and as an unadulterated by-product of the fear response itself. It would also probably be produced in a concentration or intensity that is directly correlated with the intensity of the fear experience (see de Groot et al., 2020), and regardless of other aspects of social context (e.g. the presence or absence of others).

If this is the case, then, adapting the parlance of evolutionary biologists interested in communication, emotional odours would be strictly ‘chemo-cues’ rather than ‘chemo-signals’. In other words, they may be used by receivers to infer information concerning the sender's emotional state, but they did not evolve for that communicative purpose (Maynard Smith & Harper, 2003). To date, most researchers describe emotional odours as signals, rather than cues: of the 42 primary studies we reviewed here, 22 include the words ‘chemosignal’ or ‘signal’ in their title, compared with only four (Radulescu & Mujica-Parodi, 2013; Wudarczyk et al. 2015, 2016; Zhou & Chen, 2011) that use the word ‘cue’.

We realise that not all researchers are aware of the distinction within the evolutionary biology literature, but this difference between signals and cues is an important one. It cuts to the heart of what we understand emotional odours to be and what they are for; indeed, it relates to whether they are ‘for’ anything. It may be that adopting the term ‘signal’ is appropriate: that emotional expressivity in human body odour did indeed evolve for the purpose of communicating specific emotional states and is maintained through the fitness benefits that it bestows on signallers and receivers. On the other hand, it may instead be that emotional odours are simply by-products of metabolic processes as described above, allowing inferral of the sender's emotional state by receivers but not necessarily benefiting the sender in any way (indeed, such inferral could carry costs rather than benefits, such as eliciting unwanted attention by others).

Let us therefore close this section with one brief illustration of why the distinction matters and how it might be made. Above, we argued that the adaptive utility of a fearful odour is questionable if it arrives too late to save the intended recipient from harm. Slow production of a fearful odour poses a problem if we think of it as a chemosignal, because there is a high risk of a life-critical benefit to the intended recipient(s) being missed. Yet there is no such problem if the emotional odour is a cue because there is no expected benefit and no intended recipient(s). On the other hand, a reviewer pointed out that slow production might not pose such a problem after all, because the odour could linger and thus be detected by a receiver at a later time. We agree that a slow but persistent odour could evolve as a signal. However, it could be counter-argued that such a signal would be extraordinarily reliant on favourable environmental conditions in order to persist at concentrations for long enough to be detected, and that, by then, such information would probably be out-of-date – for example, the source of the threat may have dissipated or disappeared altogether. Furthermore, the sender would have little control over who received the signal and how they responded to it, and it is therefore unclear how the signaller would benefit from producing the signal, and hence, how it could evolve or be maintained. One could speculate further, but our main points here are that (a) we do not yet know whether emotional odours are signals or cues, (b) we cannot distinguish between signals and cues until we understand more about mechanisms and the action of odours in realistic contexts, and (c) until we can make this distinction clearly, researchers should be much more circumspect about assuming odours are signals and about the kinds of social benefits that they might provide to the sender.

## Conclusion

‘It may not be a literary hyperbole that the detective hero entering a dark alley whispers, “Stay back. I smell fear, there is danger ahead”. A surprising number of people should be able to do this.’ So wrote Haviland-Jones et al. (2016: 11) in their study of how people can, with practice, get much better at discriminating emotional odours.

As keen as we are to highlight the human capacity for olfaction, however, we wonder whether this claim might itself be hyperbole. We outlined several reasons why it might be premature to claim that we can actively and accurately communicate emotional state via odours in realistic scenarios such as the fictional dark alley, let alone identify the specific emotion being communicated. Studies have produced evidence to suggest that we do emit and respond to body odours that are characteristic of specific emotional states, but these studies increase the chance of detecting such effects through various means, such as by placing strict controls on stimulus collection and participant selection, and by presenting odours in highly controlled settings. Even so, effect sizes are small to moderate, and many people appear unable to identify or label odours by emotion, at least in tested populations.

Whether the design decisions mentioned above are reasonable and appropriate depends on the scope of individual studies and the precise research questions being addressed. If the study asks whether humans are at least capable of inferring emotions from the body odour of other individuals, then each of these decisions are defendable and can be justified – controlling for sources of additional variability in the data, beyond that related to the specific hypothesis under examination, is, after all, standard scientific practice. However, this is quite different from asking whether the perception of emotional odours takes place in the absence of these control and selection procedures. Even if people really do have the *capacity* to use emotional odours in more realistic scenarios, we do not yet know whether they *actually* do so. It may be that odour plays little role in most contexts because the same information might be more quickly or efficiently communicated by other sensory means (on the other hand, it is also possible that the presence of these other cues may enhance the overall strength of response compared with any given response to a unimodal cue).

Despite our call for caution, we think that the potential for ‘smelling emotions’ is intriguing and fascinating. We outline some key areas for future research and we look forward to discoveries that follow. We particularly anticipate new discoveries of the chemical underpinnings of distinct emotional odour and of how cues of emotional odours may influence behaviour under more realistic experimental scenarios, and in people who are less ODD, than the studies reported so far.

## Research transparency and reproducibility

Data availability: the data represented in Figures 1 and 2 are taken from Calvi et al. (2020) but may also be requested from the corresponding author.
